# Editorial: New insights into fibrotic signaling in cancer

**DOI:** 10.3389/fonc.2024.1369457

**Published:** 2024-02-02

**Authors:** Patrick Ming-Kuen Tang, Eric W-F. Lam, Francis Mussal, Dongmei Zhang, Chunjie Li

**Affiliations:** ^1^ Department of Anatomical and Cellular Pathology, State Key Laboratory of Translational Oncology, The Chinese University of Hong Kong, Hong Kong, Hong Kong SAR, China; ^2^ Sun Yat-sen University Cancer Center, State Key Laboratory of Oncology in South China, Collaborative Innovation Center for Cancer Medicine, Guangdong, China; ^3^ Paediatric Oncology, Birmingham Children’s Hospital, University of Birmingham, Birmingham, United Kingdom; ^4^ College of Pharmacy, Jinan University, Guangzhou, China; ^5^ Department of Head and Neck Oncology, West China Hospital of Stomatology, Sichuan University, Chengdu, Sichuan, China

**Keywords:** cancer, tumor microenvironment, fibrotic signaling, cancer therapy, macrophage-myofibroblast transition (MMT), macrophage to Neuron-like cell Transition (MNT)

Cancer is still a top leading cause of death worldwide. Ineffective treatments, severe side effects, drug resistance, recurrence, and metastasis are major barriers to curing cancers with the conventional therapeutic methods. Immunotherapies show promise on blood cancers, but various response rates were observed on the treated patients with solid cancers, 30% of patients with non-small-cell lung cancer response to the T-cell based therapies including CAR-T and PD-1/L1 blockades in clinics.

Increasing evidence shows the importance of fibrotic signaling in solid cancers. For example, TGF-beta, a well-documented fibrotic cytokine, was first discovered as an anticancer regulator in cancer cells, but it has been found as a strong immunosuppressor for the host anticancer immunity (1, 2). Therefore, better understanding the potential roles of the fibrotic signaling in cancer cells as well as their microenvironment may eventually identify suitable strategies for improving the efficiency of cancer therapies in clinics.

This Research Topic serves as an interactive platform for sharing the latest insights of molecular mechanisms, translational potential, and clinical observations of the fibrotic singling in cancer. We have received manuscripts from research groups all over the world, and eventually accepted 6 original research and 4 review articles for publication. The articles widely covered findings from the clinical prognostic, molecular mechanisms, and therapeutic development based on the cancer cells as well as the tumor microenvironments.

## Clinical discovery

The importance of fibrotic signaling in diseases beyond tissue fibrosis has been recognized. For examples, a well-documented phenomenon “Macrophage-Myofibroblast Transition” has been widely reported in kidney fibrosis (3), its implications in solid cancer has been started to be investigated nowadays (4). In this Research Topic, the clinical implications of fibrotic signaling in clinical solid cancers have been examined, where Chen et al. observed that intratumorally fibrosis and pseudo-capsule fibrosis are positively correlated to the disease progression of renal cell. While Shan et al. observed 10 fibrotic signaling (e.g. TLR4, Hedgehog, TGF-β, etc) are closely related to the biological activities of hepatocellular carcinoma cells after reviewing 264 related studies. Qin et al. has developed a new prognostic nomogram combined with desmoplastic reaction for predicting the progression of synchronous peritoneal metastasis in colorectal cancer patients.

## Molecular mechanism

Fibrotic signaling may contribute to pathogenesis that are beyond the end stage organ diseases. Myofibroblast formation is one of the critical steps for tissue scarring (5, 6) as well as tumor formation (4). Zhang et al. reported a pathogenic role of cancer-associated fibroblasts in the irradiation driven cancer progression. Cheng et al. further summarized the clinical implications of a myofibroblast-based targeting strategies for pancreatic cancer treatment. Besides, by transcriptome profiling of patient biopsies, Tulalamba et al. identified a Wnt signaling mediator FZD10 as potential biomarker for nasopharyngeal carcinoma recurrence and Chen et al. examined the clinical relevance of FGFR signaling in head and neck carcinoma. Sukphokkit et al. demonstrated a 3-dimensional culture system which can reformed the phenotype of cholangiocarcinoma cells compared to the conventional 2D system, may serve as an ideal platform for elucidating the underlying mechanisms as well as clinical potential of fibrotic signaling in cancer *in vitro*.

## Therapeutic development

**Figure 1:**
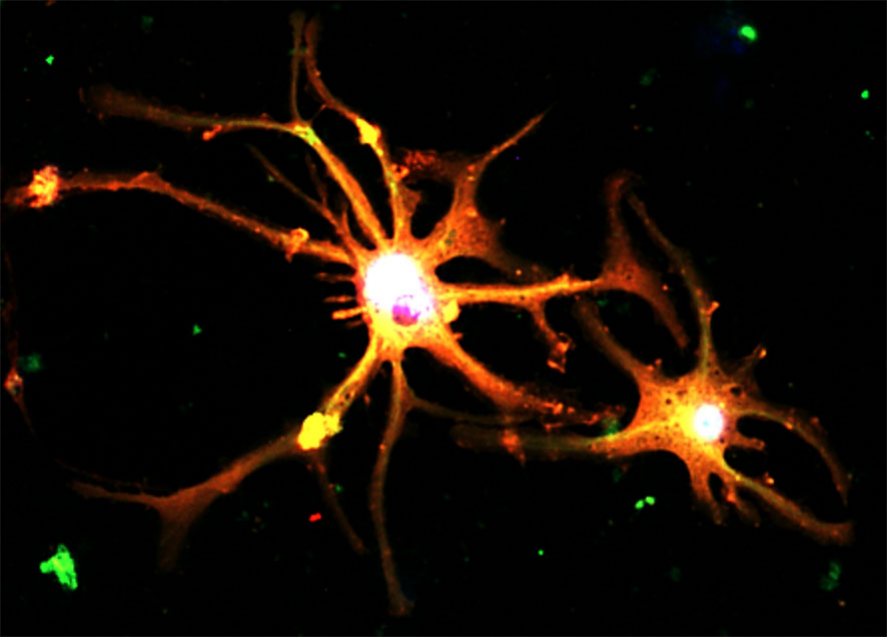
Cancer pain associated neurons formed by a TGF-β1-driven novel phenomenon “Macrophage to Neuron-like Cell Transition” in tumor microenvironment, expressing neuronal markers Synaptophysin (red) and Tubb3 (green) detecting by immunofluorescence *in vitro* (8).

Indeed, a new study demonstrated that targeting of TGF-β/Smads signaling with a natural compound formula effectively overcome drug resistance of liver cancer cells via suppressing a multidrug resistant gene MDR1 (7). Here, Gao et al. reported Icaritin, an active component of the traditional Chinese herb Epimedium genus can inhibit cancer migration via targeting Akt/mTOR signaling of the cisplatin-resistant ovarian cancer cells *in vitro*. Ji et al. summarized the dynamics and therapeutic potentials of tumor-associated macrophages in solid cancer, such as a novel neuron type derived from “Macrophage to Neuron-like Cell Transition” in lung cancer (**Figure 1**) (8). Single-cell RNA-sequencing allows researchers to dissect the TME in a cell-type specific manner, therefore the contributions of fibrotic signaling in the cancer immunity which was hidden in the conventional bulk sequencing at population level can be unmasked (9).

In conclusion, this Research Topic gathered the latest findings about fibrotic signaling in solid cancer, highlighting their clinical relevance and translational potential beyond fibrosis. We hope the collected articles can inspire both pre-clinical and translational researchers, clinical strategy targeting fibrotic signaling may eventually be developed for cancer therapy.

## Author contributions

PT: Conceptualization, Funding acquisition, Resources, Visualization, Writing – original draft, Writing – review & editing. EL: Validation, Writing – review & editing. FM: Writing – review & editing. DZ: Writing – review & editing. CL: Writing – review & editing.

## References

[1] Chan MK Chung JY Tang PC Chan AS Ho JY Lin TP. TGF-beta signaling networks in the tumor microenvironment. Cancer Lett (2022) 550:215925. doi: 10.1016/j.canlet.2022.215925 36183857

[2] Chung JY Chan MK Li JS Chan AS Tang PC Leung KT. TGF-beta signaling: from tissue fibrosis to tumor microenvironment. Int J Mol Sci (2021) 22(14):7575. doi: 10.3390/ijms22147575 PMC8303588 34299192

[3] Tang PC Chan AS Zhang CB Garcia Cordoba CA Zhang YY To KF. TGF-beta1 signaling: immune dynamics of chronic kidney diseases. Front Med (Lausanne) (2021) 8:628519. doi: 10.3389/fmed.2021.628519 33718407 PMC7948440

[4] Tang PC Chung JY Xue VW Xiao J Meng XM Huang XR. Smad3 promotes cancer-associated fibroblasts generation via macrophage-myofibroblast transition. Adv Sci (Weinh) (2022) 9:e2101235. doi: 10.1002/advs.202101235 34791825 PMC8728853

[5] Tang PM Zhang YY Xiao J Tang PC Chung JY Li J. Neural transcription factor Pou4f1 promotes renal fibrosis via macrophage-myofibroblast transition. Proc Natl Acad Sci U.S.A. (2020) 117:20741–52. doi: 10.1073/pnas.1917663117 PMC7456094 32788346

[6] Tang PM Zhou S Li CJ Liao J Xiao J Wang QM. The proto-oncogene tyrosine protein kinase Src is essential for macrophage-myofibroblast transition during renal scarring. Kidney Int (2018) 93:173–87. doi: 10.1016/j.kint.2017.07.026 29042082

[7] Chung JY Chan MK Tang PC Chan AS Chung JS Meng XM. AANG: A natural compound formula for overcoming multidrug resistance via synergistic rebalancing the TGF-beta/Smad signalling in hepatocellular carcinoma. J Cell Mol Med (2021) 25:9805–13. doi: 10.1111/jcmm.16928 PMC8505848 34514726

[8] Tang PC Chung JY Liao J Chan MK Chan AS Cheng G. Single-cell RNA sequencing uncovers a neuron-like macrophage subset associated with cancer pain. Sci Adv (2022) 8:eabn5535. doi: 10.1126/sciadv.abn5535 36206343 PMC9544324

[9] Tang PC Chan MK Chung JY Chan AS Zhang D Li C. Hematopoietic transcription factor RUNX1 is essential for promoting macrophage-myofibroblast transition in non-small-cell lung carcinoma. Adv Sci (Weinh) (2023) 11(1):e2302203. doi: 10.1002/advs.202302203 37967345 PMC10767400

